# Supramolecular interactions in the solid state

**DOI:** 10.1107/S2052252515014608

**Published:** 2015-09-22

**Authors:** Giuseppe Resnati, Elena Boldyreva, Petra Bombicz, Masaki Kawano

**Affiliations:** a Department of Chemistry, Materials, Chemical Engineering, Politecnico di Milano, 7, via Mancinelli, Milan, Lombardy I-20131, Italy; b Institute of Solid State Chemistry, Russian Academy of Sciences, ul. Kutateladze 18, Novosibirsk 128, Russian Federation; c Novosibirsk State University, ul. Pirogova 2, Novosibirsk 630090, Russian Federation; d Research Group of Chemical Crystallography, Institute of Organic Chemistry, Research Centre for Natural Sciences, Hungarian Academy of Sciences, Magyar Tudósok körútja 2, POB 286, Budapest H-1117, Hungary; e Division of Advanced Materials Science, Pohang University of Science and Technonlogy, 77 Cheongam-Ro Nam-Gu, Pohang 790-784, South Korea

**Keywords:** intermolecular interactions, crystal engineering, self-assembly, halogen bonding, polymorphism, kinetic assembly, coordination polymers, non-ambient conditions, phase transitions

## Abstract

In the last few decades, supramolecular chemistry has been at the forefront of chemical research, with the aim of understanding chemistry beyond the covalent bond. Since the long-range periodicity in crystals is a product of the directionally specific short-range intermolecular interactions that are responsible for molecular assembly, analysis of crystalline solids provides a primary means to investigate intermolecular interactions and recognition phenomena. This article discusses some areas of contemporary research involving supramolecular interactions in the solid state. The topics covered are: (1) an overview and historical review of halogen bonding; (2) exploring non-ambient conditions to investigate intermolecular interactions in crystals; (3) the role of intermolecular interactions in morphotropy, being the link between isostructurality and polymorphism; (4) strategic realisation of kinetic coordination polymers by exploiting multi-interactive linker molecules. The discussion touches upon many of the prerequisites for controlled preparation and characterization of crystalline materials.

## Abstract 2

Supramolecular interactions in the solid state are discussed in the context of crystal engineering. Specific topics include halogen bonding, ambient and non-ambient conditions, isostructurality and polymorphism, and kinetic assembly of coordination polymers.

## 1. Introduction   

Supramolecular chemistry is a highly interdisciplinary field of science covering chemical, physical and biological areas (Desiraju, 1996 ▸; Schneider & Dürr, 1991 ▸; Atwood & Steed, 2004 ▸; Steed & Atwood, 2009 ▸). It encompasses the study of crystals with all of the implied applications in the fields of solid-state chemistry, crystal engineering, catalysis and material science, including organic, inorganic, bio-organic and bio-inorganic chemistry to chemistry at interfaces, transport phenomena, polymer sciences, molecular sensors, molecular switches *etc.* (Lehn, 1988 ▸). Most bottom-up approaches to nanotechnology are based on supramolecular chemistry. Molecular self-assembly also allows the construction of non-crystalline structures such as micelles, membranes, vesicles, liquid crystals *etc.* (Ariga *et al.*, 2008 ▸). Such large structures composed of small molecules can be readily accessed through supramolecular means, requiring fewer steps of synthesis (Steed & Gale, 2012 ▸). Biological systems are often the inspiration for supramolecular research, as the study of non-covalent interactions is crucial to our understanding of many biological processes. Exploring secondary interactions is also important for the development of new pharmaceutical therapies by understanding the interactions at drug binding sites, in protein–protein interactions and also in drug encapsulation and targeted release mechanisms (Bertrand *et al.*, 2011 ▸). The overall aim is to design and develop new compounds and materials with specific new properties.

A crystal bears the collective properties of molecules moderated by intermolecular interactions. *Vice versa*, intermolecular interactions and recognition phenomena should be understandable in the context of crystal packing (Desiraju, 1996 ▸). Crystallization itself is an impressive example of molecular recognition, wherein a wide range of attractive and repulsive forces direct the formation of non-covalent bonding interactions (Dubey *et al.*, 2014 ▸). Knowledge of molecular recognition and self-assembly can also be useful to control reactive species, both in solution and in the solid state, in order to pre-organize them for a chemical reaction (Lehn, 1990 ▸). In this context, molecular recognition may be considered to be supramolecular catalysis in that non-covalent bonds hold reactive sites close together to facilitate a desired covalent synthesis. Supramolecular pre-organization of this type is especially useful when the desired reaction conformation is thermodynamically or kinetically unlikely; it may minimize side reactions, lower the activation energy for the reaction and produce the desired stereochemistry.

In crystal engineering it is crucial to distinguish different interaction types in any design strategy. It is immensely difficult to predict crystal structures of molecular substances *ab initio* because the determining factors, namely the intermolecular interactions, are weak and numerous with limited directionality (Desiraju, 2002 ▸). However, our ability to understand and quantify intermolecular interactions and the efficiency of computational methods applying supramolecular algorithms have developed considerably in the last decade, increasing significantly the long-term prospects of crystal structure prediction. Prediction of crystal structures is important both in research and industry, especially for pharmaceuticals and pigments, where understanding polymorphism is highly beneficial.

Free energy differences between polymorphs of molecular compounds are usually quite small and have different temperature dependences. A metastable crystal form can persist for a long time, or it can undergo transformation to a more stable form; in supramolecular terms, the latter constitutes an isomerization reaction. Defects in ordered crystal structures change locally the pattern of intermolecular interactions, and thus play a vital role in solid–solid phase transitions. In phase transformations, cooperativity is the essence, and structural information is transmitted through supramolecular interactions. Within a crystal, every displacement of a molecule from its equilibrium conformation, position and orientation is communicated to its immediate neighbours and hence to more distant neighbours. In a liquid, there is no such long-range correlation between molecular positions and orientations, only local effects. Polymorphic transitions in the solid state are associated with changes in molecular packing arrangements. With conversion of the supramolecular interaction pattern there can often be alterations in molecular conformations as well (Gavezzotti, 2013 ▸; Cruz-Cabeza & Bernstein, 2014 ▸). Supramolecular interactions may also alter their characteristics under non-ambient conditions (Katrusiak, 2008 ▸; Boldyreva, 2008 ▸), or be dynamic in nature.

With this incredible variety and complexity of supramolecular interactions, the potential scope of this *Feature Article* is immensely broad. The content is derived from a session held at the 23rd IUCr Congress in Montreal, which focused on a few specific themes selected to represent contemporary areas of research involving supramolecular interactions in the solid state. The topics covered are: (1) the evolution of halogen bonding to its current status as a controllable and exploitable interaction in supramolecular chemistry and crystal engineering; (2) experimental studies under non-ambient conditions to investigate the nature and balance of intermolecular interactions in the solid state; (3) the concept of morphotropy as a link between isostructurality and polymorphism, and its relationship to local intermolecular interactions; (4) controlled preparation of kinetic coordination frameworks by exploiting multi-interactive ligands. We feel that many of the prerequisites for an improved understanding of preparing and characterizing crystalline supramolecular systems are touched upon in this article.

## 2. Discussion   

### 2.1. The halogen bond: a 200 year-old story   

**Figure 1:**
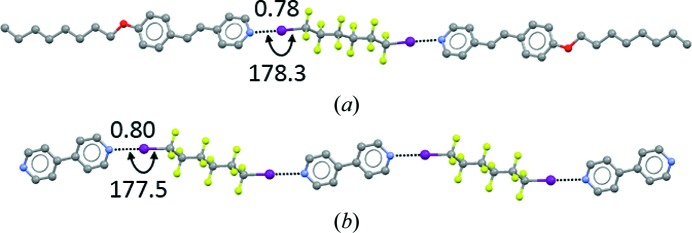
Halogen-bonded adducts formed by the bidentate XB donor 1,6-diiodoperfluorohexane. (*a*) Trimeric adduct formed with the monodentate XB acceptor bis(4-*n*-octyloxystilbazole) (Metrangolo *et al.*, 2006 ▸); (*b*) one-dimensional chain formed with the bidentate XB acceptor 4,4′-bipyridine (Cardillo *et al.*, 2000 ▸). H atoms are omitted. XBs are shown as black dotted lines. XB separations are given as values normalized to the sum of the van der Waals radii of the atoms involved and angles are given in degrees. Colour code: grey, C; light green, F; violet, I; red, O; blue, N.

**Figure 3:**
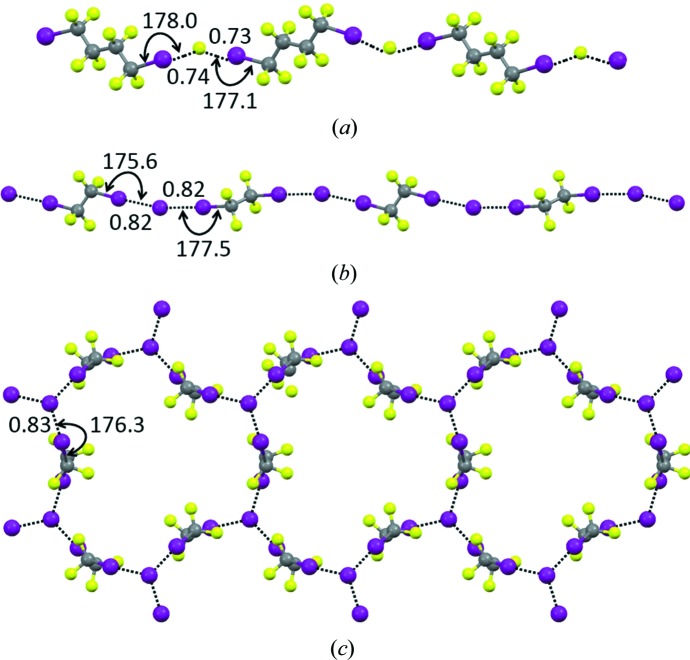
One-dimensional chains formed by bidentate XB donors: (*a*) 1,4-diodoperfluorobutane with (dimethylamino)sulfonium fluoride (Farnham *et al.*, 1988 ▸); (*b*) 1,2-diidotetrafluoroethane with tetra-*n*-butylammonium iodide (Shen & Jin, 2011 ▸). Both anions function as bidentate XB acceptors. (*c*) Two-dimensional honeycomb network formed by 1,2-diidotetrafluoroethane with iodide, acting as a tridentate XB acceptor (Liantonio *et al.*, 2006 ▸). K^+^ cations, cryptated by K222, have been omitted for clarity, although they play a major role in determining the number of XBs formed by the anion. XBs are shown as black dotted lines. XB separations are given as values normalized to the sum of the van der Waals radius of I and the Pauling ionic radius of the anion. Angles are given in degrees. Colour codes: grey, C; light green, F; violet, I.

Preparation of the I_2_⋯NH_3_ adduct was described as early as 200 years ago (Colin, 1814 ▸), but the potential of halogen atoms to drive recognition phenomena and self-assembly processes long remained unrecognized. The general and manifold ability of halogen atoms to be involved in attractive interactions was acknowledged only in the late 1990s when it was observed that metal-bound Cl often accepts hydrogen bonds (Aullón *et al.*, 1998 ▸) and that the I atom of iodoperfluorocarbons gives quite strong interactions with atoms possessing lone pairs (Metrangolo & Resnati, 2001 ▸; Figs. 1 ▸ and 2 ▸) or with anions (Cavallo *et al.*, 2010 ▸; Fig. 3 ▸). This latter type of interaction, where the halogen atom acts as an electrophile (as in the I_2_⋯NH_3_ adduct mentioned above), has now become a valuable tool in crystal engineering. According to a recent IUPAC recommendation, these interactions are named halogen bonds (Desiraju *et al.*, 2013 ▸). Specifically: ‘*A halogen bond occurs when there is evidence of a net attractive interaction between an electrophilic region associated with a halogen atom in a molecular entity and a nucleophilic region in another, or the same, molecular entity*’. Throughout this article, the acronym XB will be used to denote *halogen bond*.

**Figure 4:**
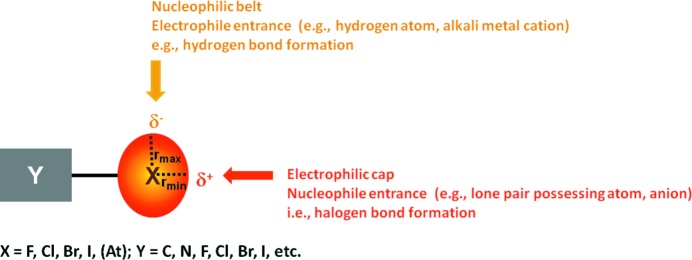
Schematic representation of the anisotropic distribution of the electron density around a monovalent halogen atom and the pattern of the resulting interactions.

In organic compounds, halogen atoms are normally at the periphery of molecules, so their positions are particularly appropriate to be involved in non-covalent interactions. Halogen atoms have a relatively high electronegativity and in halo-organics they are commonly understood to be sites of high electron density that can function as electron donors (nucleophiles), *e.g.* when coordinating H atoms or alkali metal cations (Metrangolo & Resnati, 2013 ▸). However, the electron density in covalently bound halogen atoms is anisotropically distributed (Politzer *et al.*, 2013 ▸). In monovalent halogen atoms, there is a region of higher electron density which forms a negative belt orthogonal to the covalent bond involving the halogen atom. Nucleophiles approach the halogen in this region, which accounts for the observed directionality in the coordination of H atoms and alkali metal cations (Fig. 4 ▸). A region of lower electron density is present on the elongation of the covalent bond formed by the halogen. It generates a cap (the so-called σ-hole) where the electrostatic potential is frequently positive (mainly in the heavier halogens) so that attractive interactions develop with electron-rich sites. Persistent biases resulting from the commonplace approximation that halogen atoms are neutral spheres in dihalogens and negative spheres in halocarbons long prevented recognition of this amphoteric character as a general feature of halogenated derivatives. Positive, namely electrophilic, halogens appeared as an exception (Politzer *et al.*, 2010 ▸) and it was not acknowledged how strong interactions given by electrophilic halogens can be. The very minor attention given to halophilic reactions (Grinblat *et al.*, 2001 ▸; Foucaud, 1983 ▸) was probably another consequence of the same biases.

While the interest in interactions formed by electrophilic halogens has grown rapidly over the last 15 years, the XB practice and concept developed through a rather patchy course. Various reviews appeared on the topic with specific focus on modelling and computation (Politzer *et al.*, 2013 ▸), conventional (Metrangolo *et al.*, 2008 ▸) and unconventional (Troff *et al.*, 2013 ▸) tectons in crystalline systems, anion coordination in solids (Metrangolo *et al.*, 2009 ▸) and in liquids (Beale *et al.*, 2013 ▸), molecular materials (Priimagi *et al.*, 2013 ▸) and drug–receptor binding (Parisini *et al.*, 2011 ▸). However, a coherent historical perspective on the interaction has so far been lacking. A description of how the concept emerged and became accepted by a broad chemical community should complement the reviews listed above and may offer an alternative way to understand the general features of the interaction and its potential in crystal engineering (Cavallo *et al.*, 2014 ▸). The object here is to summarize papers which reported major experimental findings in the field or which gave important theoretical contributions for development of the XB concept. Particular attention is paid to XB in the solid state, in accordance with the overarching theme of this article.

#### 2.1.1. A historical perspective   

The I_2_⋯NH_3_ adduct, probably the first halogen-bonded system ever prepared, was synthesized as a liquid with a somewhat metallic luster in J. L. Gay-Lussac’s laboratory by J. J. Colin as early as 1813 (Colin, 1814 ▸). Fifty years later, Guthrie obtained the same liquid in pure form by adding powdered I_2_ to aqueous ammonia and proposed that the formed compound had the structure NH_3_·I_2_ (Guthrie, 1863 ▸). Anions were also soon discovered to interact attractively and to form adducts with halogen atoms. I_3_^−^, specifically strychnine triiodide, was the first prepared species of the class of compounds formed by reaction of anions (the nucleophile, XB-acceptor, in this case I^−^) with dihalogens (the electrophile, XB-donor, in this case I_2_) (Pelletier & Caventou, 1819 ▸). Also, the greater solubility of I_2_ in different solvents on addition of metal iodides attracted early attention (Svensson & Kloo, 2003 ▸). While numerous investigators suggested that these observations were rationalized by the formation of I_3_^−^, others were reluctant to accept this explanation and in 1870 the first systematic investigation on the topic was published (Jörgensen, 1870 ▸). Halocarbons were reported to give adducts similar to those formed by dihalogens only in 1883 when the quinoline/iodoform crystalline adduct, probably the first halogen-bonded adduct prepared from a halocarbon, was described (Roussopoulos, 1883 ▸).

Bromine and chlorine were reported to form halogen-bonded adducts similar to iodine only at the end of the 19th century when the 1:1 dimers formed by Br_2_ and Cl_2_ with various amines were described (Remsen & Norris, 1896 ▸). This timeline of adduct description is consistent with the fact that the XB-donor ability is greater for the heavier and more polarizable halogens, namely it increases in the order Cl < Br < I (Bertani *et al.*, 2010 ▸; Politzer *et al.*, 2010 ▸). Fluorine is the least heavy and polarizable halogen, and therefore the least prone to function as an XB donor (Metrangolo *et al.*, 2011 ▸), and the first F_2_–neutral-nucleophile adducts (*e.g.* F_2_⋯NH_3_ and F_2_⋯OH_2_) were reported only in the 1990s (Legon, 1999 ▸). F_3_^−^ (namely F_2_⋯F^−^ if the XB notation is used) was first observed in 1976 (Riedel *et al.*, 2010 ▸), and extreme conditions (low temperatures and pressures) were required for its isolation. Astatine is the heaviest halogen and its polarizability has been calculated to be higher than that of iodine (Schwerdtfeger, 2006 ▸). To the best of our knowledge, no halogen-bonded adduct has been reported for this element to date, but astatine is expected to function as an XB donor even more effectively than iodine. This expectation is supported by computational results (Alkorta *et al.*, 2008 ▸).

**Figure 5:**
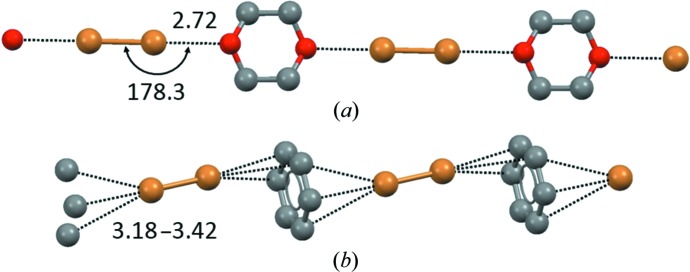
One-dimensional chains formed by Br_2_ (working as a bidentate XB donor) with 1,4-dioxane (*a*) and benzene (*b*), both working as bidentate XB acceptors. H atoms are omitted. XBs are shown as black dotted lines. Distances are given in Å and angles in degrees. Colour code: grey, C; red, O; brown, Br.

Description of various observations and phenomena where we now recognize the role played by XB went on through the entire 20th century. Most of the important discoveries reported in the last 70 years are summarized below. In 1948, UV–vis spectroscopy allowed the I_2_–benzene complex to be identified in solution and 1 year later other aromatics were reported to behave analogously (Benesi & Hildebrand, 1949 ▸). R. S. Mulliken described in 1950 the formation of similar complexes with ethers, thioethers and carbonyl derivatives (Mulliken, 1950 ▸) and 2 years later he rationalized them as a subclass of the electron donor–acceptor molecular complexes (Mulliken, 1952 ▸). The appearance in UV–vis spectra of bands specific for charge transfer from the electron-density donor to the halogen atom was shown by complexes involving dihalogens and aromatics (Rosokha & Kochi, 2008 ▸) and by many other halogen-bonded adducts, even as weak as the perfluoro­carbon/amine complexes (Burdeniuc *et al.*, 1998 ▸). The Br_2_⋯O(CH_2_CH_2_)_2_O adduct was the first reported X-ray structure of a halogen-bonded system (Hassel *et al.*, 1954 ▸; Fig. 5 ▸ *a*) and several related crystal structures of adducts involving dihalogens and halocarbons were then established in rapid sequence (Hassel, 1970 ▸). The crystal structures of Br_2_⋯C_6_H_6_ (Fig. 5 ▸ *b*) and Cl_2_⋯C_6_H_6_ (Hassel *et al.*, 1959 ▸) are particularly noteworthy as they proved that π-systems work as donors of electron density to electrophilic halogens also in the solid state (Vasilyev *et al.*, 2001 ▸). Importantly, these systems suggested that halogen-bonded adducts are on the reaction pathways of halogenation reactions of aromatics and other unsaturated systems. In the successive decades, this hypothesis was forcefully confirmed (Lenoir & Chiappe, 2003 ▸) and it was shown that π-donating units form solid adducts also with halocarbons (Rosokha & Kochi, 2008 ▸).

In 1968, a comprehensive review by Bent analysed the structural chemistry of donor–acceptor adducts, and halogen-bonded systems were included (Bent, 1968 ▸). This review showed the main geometric feature of the XB in the solid state, namely linearity, and this feature was successively validated by statistical analysis of the Cambridge Structural Database (CSD; Desiraju & Parthasarathy, 1989 ▸). Another review in the early 1980s collected consistent indications afforded by several techniques (*e.g.* UV–vis, IR and Raman, NMR and NQR, dielectric polarization) and proved that the interaction occurs in the liquid phase as well as in the solid state (Dumas *et al.*, 1983 ▸).

At the end of the 20th century, microwave spectroscopy of halogen-bonded adducts in the gas phase (Legon, 1999 ▸) showed that the interaction in ‘isolated’ adducts is largely the same as in adducts in condensed phases, *i.e.* the solvent and lattice effects typical for liquids and solids do not have any major influence on the interaction characteristics. In the same period, we proved systematically that halocarbons and anions form adducts (Cavallo *et al.*, 2010 ▸; Fig. 3 ▸) similar to those formed by halocarbons with heteroatoms possessing lone pairs (Metrangolo *et al.*, 2005 ▸; Figs. 1 ▸ and 2 ▸). We also expanded the range of halocarbons that work as effective XB donors (Lunghi *et al.*, 1998 ▸) and revealed the key role that residues close to halogen atoms have in determining the strength of the formed XBs. The fine tuning of the structural and functional features of adducts formed under XB control became possible *via* an appropriate choice of the nature and structure of the involved tectons.

In the middle of the 1980s, a statistical analysis of crystal structures in the CSD disproved the approximation that halogen atoms in halocarbons are spherical (Nyburg & Faerman, 1985 ▸). It showed that they have an ellipsoidal shape with a shorter radius (*r*_min_) on the extension of the covalent C–halogen bond and a longer radius (*r*_max_) orthogonal to this direction (Fig. 4 ▸). A few years later, the electrostatic potential on the surface of monovalent halogen atoms was calculated, revealing an anisotropic distribution of the electron density (Brinck *et al.*, 1992 ▸). The region of most negative potential, forming a belt orthogonal to the covalent bond, and the region of most positive potential, forming a cap on the extension of the covalent bond, correspond respectively to the shorter and longer radii identified *via* the CSD search. These two findings gave the experimental and theoretical basis for the comprehensive process of unification of all observations related to electrophilic halogens. First, a single and unified model was put forward (Metrangolo & Resnati, 2001 ▸), together with a comprehensive recollection of the observations summarized above and many others not mentioned here. A subsequent review article (Metrangolo *et al.*, 2005 ▸) further developed the unified understanding of previously unrelated phenomena. While acknowledging that differences exist in adducts formed when dihalogens, halocarbons or other halogenated derivatives attractively interact with atoms possessing lone pairs, or π-systems, or anions, it also underlined that the main chemical and physical features of the formed adducts remain largely the same.

### 2.2. Varying pressure to study interactions in the solid state   

A common approach to the study of supramolecular interactions is to analyse a crystal structure at ambient conditions. With the development of hardware and software for non-ambient crystallography, variable temperature/pressure have become increasingly common as tools to study supramolecular interactions in chemical, biomimetic and biological systems (Boldyreva & Dera, 2010 ▸; Lee *et al.*, 2014 ▸; Machon *et al.*, 2014 ▸). Diffraction studies carried out at elevated pressures are increasing in popularity. Usually pressures do not exceed 10 GPa due to the hydrostatic limits of pressure-transmitting liquids (Angel *et al.*, 2007 ▸; Balla Ballaran *et al.*, 2013 ▸), but for organic solids this is more than sufficient to observe many interesting phenomena related to intermolecular interactions. DFT modelling of crystalline molecular systems at high pressures is also becoming widespread (Averkiev *et al.*, 2014 ▸; Bruce-Smith *et al.*, 2014 ▸; Cai *et al.*, 2014 ▸; Liu *et al.*, 2015 ▸; Macchi, 2013 ▸; Macchi *et al.*, 2014 ▸; Tse & Boldyreva, 2012 ▸; Xiang *et al.*, 2014 ▸).

All forms of non-covalent interactions – hydrogen bonds, halogen bonds, stacking interactions, van der Waals interactions *etc.* – can be characterized by their geometry (distances and angles) and energy (vibrational and optical absorption band wavenumbers, intensities, widths). A comparative analysis of multiple structures, rather than a static analysis of a single structure, can give a clue to understanding the relative role of different types of intermolecular interactions in the solid state. The ‘multiple structures’ to be compared with the ambient-pressure phase can be: (1) the same solid phase undergoing a continuous anisotropic distortion on *P*,*T* variations; (2) new solid phases formed as a result of a solid-state structural rearrangement (phase transition); (3) new solid phases crystallized from solution or from the melt under non-ambient conditions.

#### 2.2.1. Anisotropy of continuous strain   

Already in the early papers by Fedorov and Ubbelohde, data on the anisotropy of structural strain from temperature variations were used to estimate the relative strength of intermolecular interactions, with particular emphasis on hydrogen bonds. In addition, this anisotropy was used to distinguish between attractive and repulsive interactions (Fedorov, 1949 ▸; Gallagher *et al.*, 1955 ▸; Robertson & Ubbelohde, 1939 ▸; Ubbelohde, 1939 ▸; Ubbelohde & Woodward, 1946 ▸). This approach is also often used nowadays (Boldyreva *et al.*, 1997*a* ▸,*b* ▸; Boldyreva, Drebushchak *et al.*, 2004 ▸; Drebushchak & Boldyreva, 2004 ▸; Drebushchak, Kolesnik & Boldyreva, 2006 ▸; Engel *et al.*, 2014 ▸; Zhang *et al.*, 2014 ▸). A similar approach is to derive information on intermolecular interactions from data on the anisotropy of structural strain induced by hydrostatic compression. Systematic studies in this direction started in the 1990s (Boldyreva, 1994 ▸; Boldyreva, Ivashevskaya *et al.*, 2004 ▸; Boldyreva, Drebushchak *et al.*, 2004 ▸; Boldyreva *et al.*, 1994 ▸, 1997*a* ▸,*b* ▸; Boldyreva *et al.*, 1998 ▸, 2000 ▸, 2001 ▸, 2003 ▸; Drebushchak & Boldyreva, 2004 ▸; Katrusiak, 1991 ▸; Masciocchi *et al.*, 1994 ▸) and are very popular nowadays. Of special interest are examples where the anisotropy of strain on cooling and with increasing pressure is radically different, even for the same change in volume. As an extreme difference, a structure can be *compressed* in selected crystallographic directions on cooling and *expand* in the same directions on hydrostatic compression, or *vice versa* (Boldyreva *et al.*, 1998 ▸; Boldyreva, Drebushchak *et al.*, 2004 ▸; Kapustin *et al.*, 2015 ▸). In molecular crystals there are usually several different types of intermolecular interactions present, and their respective roles in forming the crystal structure can change depending on the temperature, and especially, pressure. Comparative analysis of the orientation of the principal axes of strain ellipsoids makes it possible to understand the intermolecular interactions and their structure-forming roles, as well as to compare the relative strengths of the interactions at different pressures. This topic has been reviewed in the literature (Boldyreva, Ivashevskaya *et al.*, 2004 ▸; Boldyreva, Drebushchak *et al.*, 2004 ▸; Boldyreva, 2003 ▸, 2004 ▸, 2008 ▸, 2009 ▸, 2014 ▸; Boldyreva & Dera, 2010 ▸; Boldyreva, Drebushchak *et al.*, 2004 ▸). Recent examples include analysis of pressure-induced strain in dl-serine (Zakharov *et al.*, 2012 ▸), *N*-acetyl-l-cysteine (Minkov & Boldyreva, 2013 ▸), dl-homocysteine (Minkov & Boldyreva, 2014 ▸), and in a series of methylated glycine derivatives (Kapustin *et al.*, 2014 ▸, 2015 ▸). An overview of recent activity has also been given by Hejny & Minkov (2015 ▸).

#### 2.2.2. Phase transitions   

**Figure 6:**
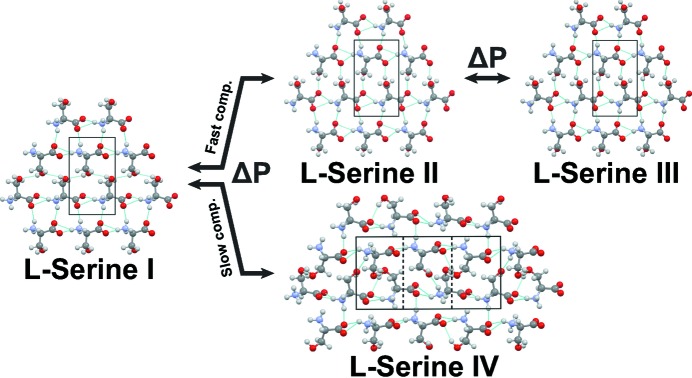
The complexity of polymorphic transitions in crystalline l-serine depending on the rate of pressure increase. Reprinted with permission from Fisch *et al.* (2015 ▸). Copyright (2015) American Chemical Society.

Another approach to study intermolecular interactions is to follow structural changes on variations of pressure. Examples where hydrogen bonds play the main role over the course of structural phase transitions are the most numerous in comparison to other types of intermolecular interactions (see, for example, Boldyreva, 2008 ▸, 2009 ▸, 2014 ▸; Boldyreva, Sowa *et al.*, 2006 ▸; Drebushchak, Sowa *et al.*, 2006 ▸; Fisch *et al.*, 2015 ▸; Hejny & Minkov, 2015 ▸; Katrusiak, 1992 ▸, 1996 ▸, 2003 ▸; Katrusiak & Nelmes, 1986 ▸; Kolesnik *et al.*, 2005 ▸; Moggach *et al.*, 2005 ▸, 2006 ▸; Zakharov & Boldyreva, 2013 ▸, 2014 ▸). This is not surprising; hydrogen bonds act as ‘springs’, enabling elastic (reversible on decompression) structural strain thereby preserving not merely crystallinity, but also the integrity of a single crystal. This is of the utmost importance for determining crystal structures of high-pressure phases. Distortion, switching over and disordering of hydrogen bonds have been shown to be related to the rotation of molecular fragments and changes in molecular conformations. A question which still remains to be answered is of a ‘chicken and egg’ type: does the increasing pressure or temperature cause the molecules to start to rotate, thus changing their conformations in order to enable a closer packing, with hydrogen bonds and other intermolecular contacts following this primary process? Or does pressure or temperature directly influence the intermolecular contacts and interactions and, after they have changed, the rest of the molecular fragments are forced to adapt? It is likely that the answer may differ for the same crystal depending on the temperature-pressure range. To add complexity, different high-pressure phases can be formed depending on the choice of pressure-transmitting fluid (Boldyreva, 2007 ▸; Boldyreva, Ahsbahs *et al.*, 2006 ▸) or on the rate of pressure increase (Fisch *et al.*, 2015 ▸; Tumanov *et al.*, 2010 ▸; Zakharov *et al.*, 2015 ▸) (Fig. 6 ▸).

Structural rearrangements with increasing pressure can also manifest from other types of non-covalent interactions. For example, crystals of α-AuEt_2_DTC·*x*CH_2_Cl_2_ exhibit highly unusual negative-area compressibility, due to the spring-like compression of helices. Above 0.05 GPa these crystals transform to the β phase, where the Au_16_-pitch helices partly unwind their turns, relaxing the tension generated by external pressure between neighbouring helices of the opposite handedness. This is a unique observation of atomic scale helical filament transformation, which is on a more macroscopic scale a universal process analogous to the helix reversal between DNA forms B and Z. In the macroscopic world it is similar to the non-periodic unwind kinks in grapevine tendrils and telephone cords. Pressure also reduces the differences between the ligand-supported and unsupported Au^+^⋯Au^+^ bonds (Paliwoda *et al.*, 2014 ▸). Some similarities can be seen between this transformation and an irreversible pressure-induced unravelling of helices into layers over the course of the γ- to δ-glycine phase transition (Boldyreva, Ivashevskaya *et al.*, 2004 ▸; Boldyreva *et al.*, 2005 ▸), which also mimics a biological process, namely the unravelling of collagen on ageing (Goryainov *et al.*, 2006 ▸). A pressure-induced phase transition in α-chlorpropamide immersed in its saturated ethanol solution almost perfectly preserves the hydrogen-bond network (hydrogen bonds slightly *expand*), but molecular conformations and other non-covalent interactions change significantly, enabling a denser molecular packing (Seryotkin *et al.*, 2013 ▸).

Pressure-induced cooperative rotation of molecular anions can also account for cooperative and reversible structural phase transitions in crystal structures without any hydrogen bonds at all. An example is provided by an isosymmetric phase transition in Na_2_C_2_O_4_ (Boldyreva, Ahsbahs *et al.*, 2006 ▸). In general, it is important to consider all interactions in a crystal structure over the course of pressure variations. Only in doing so is it possible to understand and predict pressure-induced structural changes. Selected interactions can be destabilized by pressure for overall enthalpic benefit. For example, compression of a series of Co_2_(CO)_6_(*X*Ph_3_)_2_ (*X* = P, As) crystalline compounds results in *expansion* of the Co—Co distance and the phenyl ligands adopt an eclipsed conformation instead of the staggered one observed under ambient conditions. Nevertheless, the total change in the enthalpy is favourable since the new structure enables denser packing (Casati *et al.*, 2005 ▸, 2009 ▸).

#### 2.2.3. Crystallization at non-ambient conditions   

Another opportunity to understand intermolecular interactions and their structure-forming role is to study crystallization under variable-pressure conditions. The outcome of the process is determined by the interplay of nucleation and crystal growth. Different interactions are responsible for these two processes, and the relative role of different interactions differs with increasing pressure. Therefore, variations of pressure can help to control polymorphism and to obtain metastable forms.

Most often these high-pressure approaches are used when aiming to crystallize compounds that are liquid under ambient conditions. It is known from early high-pressure experiments that the same liquid can form different polymorphs when cooled or when compressed. Examples include crystallization of benzene (Block *et al.*, 1970 ▸; Budzianowski & Katrusiak, 2006 ▸; Fourme *et al.*, 1971 ▸; Piermarini *et al.*, 1969 ▸; Raiteri *et al.*, 2005 ▸; Thiéry & Léger, 1988 ▸), ethanol, acetic acid (Allan & Clark, 1999 ▸), water (Kuhs, 2007 ▸; Pounder, 1965 ▸), acetone (Allan *et al.*, 1999 ▸), and many other small molecules. Both of the obtained polymorphs can be thermodynamically stable and their comparison allows a better understanding of the structure-forming factors and crystallization process.

Another possibility is to obtain different polymorphs by compressing the same liquid to different pressures. Again, both of the obtained polymorphs can be thermodynamically stable. However, the situation can be much more complex if kinetic factors come into play such that one or even neither of the polymorphs is in fact thermodynamically stable. In addition the sequence of accessing the different pressure points may be important, as well as the compression rate. Different polymorphs are often obtained depending on the ‘direction of approaching a selected pressure’ (*i.e.* on increasing or decreasing pressure) (Ridout *et al.*, 2014 ▸).

**Figure 7:**
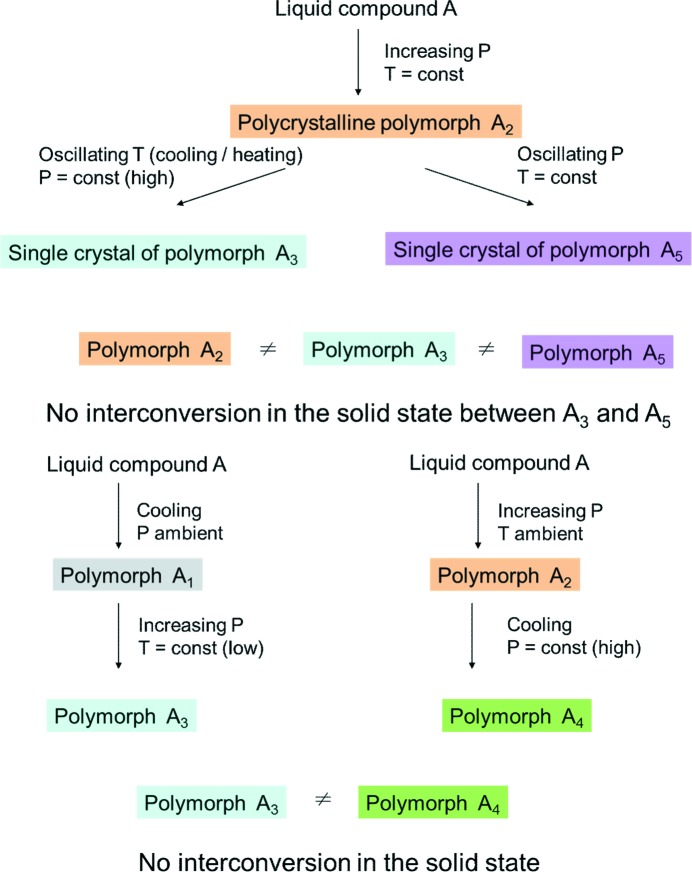
Different polymorphs formed depending on the protocol of reaching the same (*T*,*P*)-point.

The situation is additionally complicated if the aim is to obtain a high-pressure polymorph as a single crystal. Usually compression of a liquid leads to a polycrystalline sample. In order to obtain a single-crystal suitable for X-ray analysis, different strategies can be employed (Fig. 7 ▸). A polycrystalline sample at high pressure can be heated until all crystallites but one are dissolved, after which the heating is stopped and the only remaining crystallite grows. An alternative (yet more experimentally difficult, and hence more rarely used) is to keep temperature constant, but to decrease the pressure until all crystallites but one dissolve. Subsequently, pressure is increased once again to allow the only remaining crystallite to grow. Although the same final (*T*,*P*) point is reached in both experiments, their results can be completely different, with different polymorphs formed. Obviously, at least one of them should be metastable. Moreover, the different polymorphs can never interconvert in the solid state. Many examples of this type have been reported in the papers by Katrusiak *et al.*, where such effects were studied systematically (Bujak & Katrusiak, 2010 ▸; Dziubek *et al.*, 2007 ▸; Katrusiak *et al.*, 2011 ▸; Olejniczak & Katrusiak, 2010 ▸, 2011 ▸; Olejniczak *et al.*, 2010 ▸). A case study is the polymorphism of bromochlorofluoroacetic acid (Gajda *et al.*, 2009 ▸), where pressure affects the balance between secondary intermolecular interactions involving halogen and O atoms. Depending on the protocol with which pressure is increased, either catemers (phase α) or dimers (phase β) were the main structure-forming units in the polymorph formed. Another example is related to comparing molecular aggregation in pyridazine, pyridine and polymorphs of benzene (Podsiadło *et al.*, 2010 ▸). The interactions governing the molecular arrangement in the series of structures were shown to change gradually from C—H⋯N to C—H⋯π hydrogen bonds. High pressures favoured C—H⋯N interactions over the C—H⋯π bonds in pyridine, but benzene remained more stable than pyridazine and pyridine.

Another possibility to study the structure-forming role of non-covalent interactions is to consider pressure-induced crystallization of compounds that are soluble in selected fluids under ambient conditions (Fabbiani *et al.*, 2004 ▸). Also in this case crystallization results from an interplay between thermodynamic and kinetic factors and between the effect of pressure on nucleation and subsequent growth of the crystal nuclei. One possibility is to observe crystallization of the form that is thermodynamically stable at high pressure. In contrast with attempts to obtain this form *via* a direct pressure-induced solid-state transformation, starting from solution can facilitate nucleation of the new phase, thus yielding complete transformation to the high-pressure polymorph (Fabbiani *et al.*, 2004 ▸; Fabbiani & Pulham, 2006 ▸; Oswald *et al.*, 2009 ▸). If a kinetic barrier also exists, hindering transformation from the high-pressure to the ambient-pressure polymorph on decompression, then the high-pressure form can be preserved (‘quenched’) by rapid release of pressure. This ‘quenched’ crystal can then be used to seed mass crystallization at ambient conditions, for example in the pharmaceutical industry (Fabbiani *et al.*, 2009 ▸, 2014 ▸).

Another outcome may be that compression of solutions favours crystallization of form(s) that are metastable at high pressure, but nucleate faster than a thermodynamically stable form. The growth of such a phase can kinetically impede growth of the stable form. In this circumstance, it is possible that molecules of a dissolved compound adopt different conformations in solution at high pressure, as compared with those under ambient conditions. The crystal structure is then formed by aggregation of molecules in the high-pressure conformations, and the lattice energy is optimized for these molecular conformations, not for the ambient-pressure ones. Common methods of crystal structure prediction which are based on molecular conformations optimized in the gas phase may not be adequate in such cases. In particular, this approach may become troublesome when crystallization from solution is considered since the molecular conformation in solvent under pressure may be completely different.

An additional factor to consider is that crystal growth at high pressure can be hindered, such that very high values of supersaturation can be achieved. In such cases, crystallization of high-pressure forms is not observed on compression, but instead upon decompression. Rapid increase of pressure to rather high values can help to prevent crystallization even if the sample is kept at this pressure for a very long time.

A special case is the crystallization of high-pressure forms (polymorphs or solvates) on compression of solids immersed in pressure-transmitting fluids. Even if a solid is poorly soluble in the fluid under ambient conditions, its solubility can increase with increasing pressure. In this case, marked recrystallization or a seemingly solid-state (but in fact solvent-assisted) phase transition can take place. The latter can be suspected when the ‘solid-state’ pressure-induced phase transition depends on the choice of pressure-transmitting fluid: either the transition points and/or the structures of the high-pressure form differ (Boldyreva, 2007 ▸; Fabbiani *et al.*, 2005 ▸, 2007 ▸). Again, in these situations observation of the high-pressure forms can take place not on increasing pressure, but on decompression, and can be prevented by increasing pressure at a sufficient haste (Tumanov *et al.*, 2010 ▸; Zakharov *et al.*, 2015 ▸). Formation of solvates at high pressure, even for compounds that are reluctant to form solvates at ambient conditions, is another indication that intermolecular interactions in solutions are changed significantly by pressure (Fabbiani *et al.*, 2010 ▸; Olejniczak & Katrusiak, 2011 ▸). This phenomenon seems to be directly related to interactions of proteins with solvents at high pressure.

### 2.3. From isostructurality to polymorphism   

Regarding the crystal as a supramolecular entity, the emphasis is laid on the collective properties of molecules mediated by intermolecular interactions. Control of the physicochemical properties of materials can be approached by fine-tuning of the structural properties. This might be achieved by introduction of substituents on molecules or guest molecules of different sizes, shapes and chemical composition. The balance of spatial requirements and electrostatic effects ultimately determine the packing arrangement. The capability of fine-tuning requires first the recognition, then an increasingly deeper understanding of supramolecular interactions in the solid state, along with consideration of molecular, supramolecular and crystallographic symmetry. Crystal engineering is ultimately facilitated by application of this accumulated knowledge.

Supramolecular interactions control the path of molecular recognition, the set-up of the crystal packing arrangement, and in the case of flexible molecules the molecular conformation (Bombicz *et al.*, 2014 ▸). A given packing arrangement might tolerate small molecular changes while keeping the related crystals isostructural. Chemical modification can involve alteration of the position or size of a substituent or replacement of a particular constituent in a multi-component system. The flexibility of a crystal is provided by the 30–40% of free space that is inevitably present in a close-packed molecular structure. Investigation of isostructurality in crystals leads to deeper understanding of close-packing principles. In contrast to polymorphism, structural similarity can be more readily quantified by numerical descriptors (Kálmán *et al.*, 1993 ▸; Kálmán & Párkányi, 1997 ▸) such as cell similarity (π), isostructurality (*I*_s_) and molecular isometricity indices (*I*_m_).

**Figure 8:**
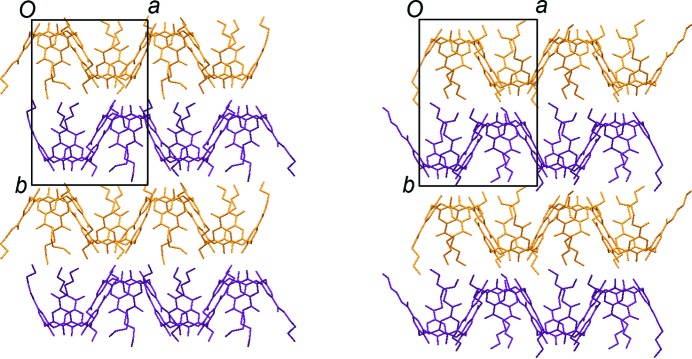
Supramolecularly initiated morphotropy. Upper-rim-site lipophilic calix[4]arenes are receptors for neutral terpenes (Gruber *et al.*, 2011 ▸). The preserved packing motif of the host molecule is in the *ac* crystallographic planes (horizontal). The planes are related by translation in the crystals of the menthol and menthone inclusion compounds.

When the tolerance within a particular crystal structure is exceeded, a different packing arrangement is developed. However, packing motifs may still be preserved, in which the original supramolecular interaction pattern is conserved. These motifs may be moved relative to each other, either by rotation or translation (Fig. 8 ▸). This phenomenon is called morphotropy, and it provides a link between isostructurality and polymorphism (Kálmán & Fábián, 2007 ▸). A morphotropic change can be initiated either by a covalent modification of the molecule or by alteration of the supramolecular interactions.

**Figure 9:**
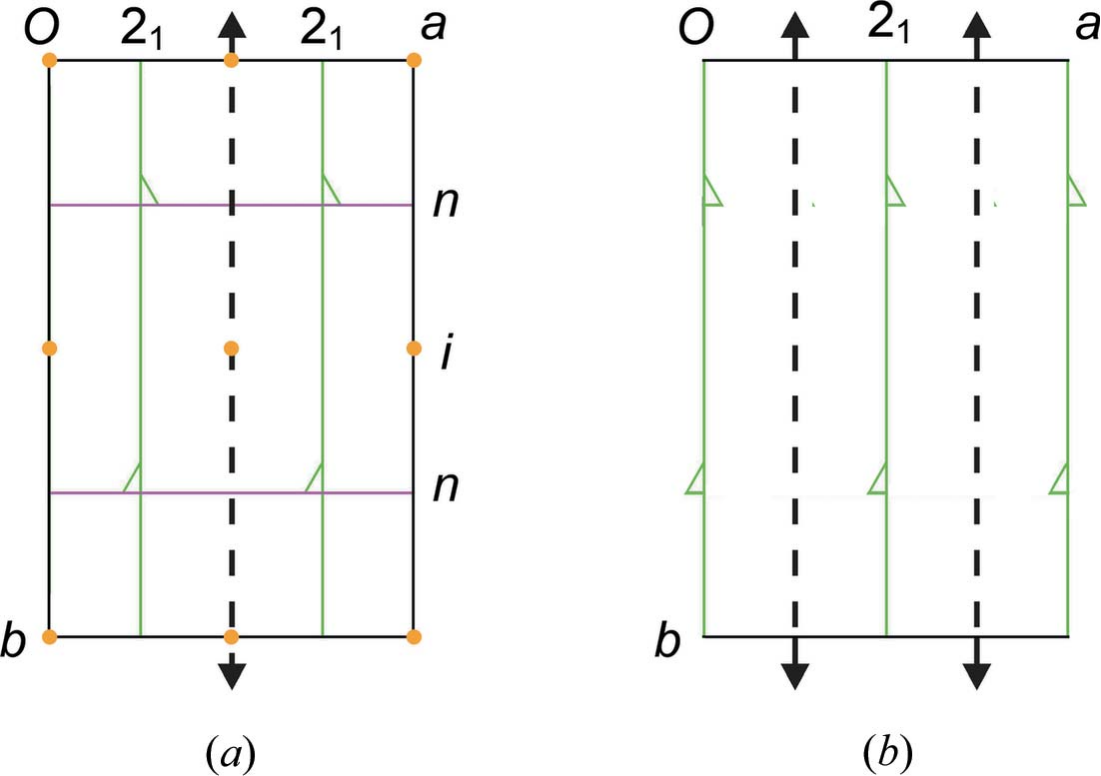
Crystal symmetries in two supramolecularly initiated morphotropic crystals where the corresponding structures are related by virtual non-crystallographic symmetries. The virtual twofold axes are indicated by dotted lines in (*a*) space group *P*2_1_/*n* (Gruber *et al.*, 2006 ▸) and (*b*) space group *P*2_1_ (Gruber *et al.*, 2011 ▸).

There is an example (Fig. 9 ▸ *a*) (Gruber *et al.*, 2006 ▸) of a series of ester-substituted dinitro calixarene inclusion compounds where the host placements in the corresponding crystals are related by a virtual, non-crystallographic twofold rotation, retaining the space group *P*2_1_/*n*. Another example is a series of upper-rim-substituted lipophilic calix[4]arenes (Fig. 9 ▸ *b*) (Gruber *et al.*, 2011 ▸), where the location of the corresponding host molecules are related by a virtual, non-crystallographic twofold rotation at *y* = ¼ and ¾ along the crystallographic *b* axis in space group *P*2_1_. The fundamental packing motifs are driven by the supramolecular interactions, and may remain even if the crystal systems and space groups are changed by the introduction of substituents or guest molecules.

**Figure 10:**
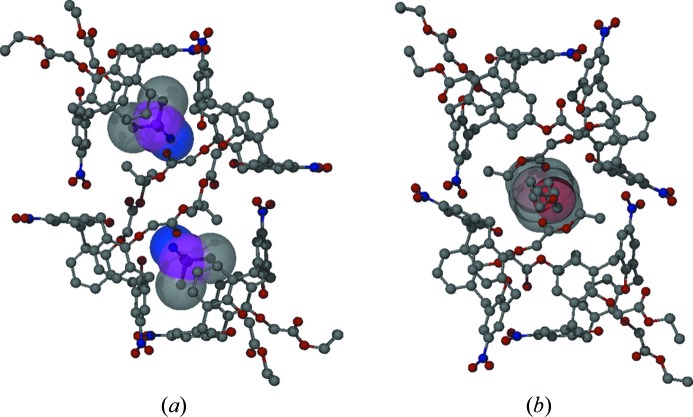
The placement of guest molecules, (*a*) in the calix crater (polar aprotic solvent, *e.g.* DMSO) or (*b*) among the lower-ring substituents (protic solvent, *e.g. n*-BuOH), determined by the supramolecular interactions in morphotropically related crystal lattices.

A series of laterally non-, mono- and disubstituted calixarenes (Fischer *et al.*, 2007 ▸, 2011 ▸, 2012 ▸, 2013 ▸; Gruner *et al.*, 2010 ▸) presents an example. Different host–guest stoichiometry is realised depending on the guest recognition modes in the upper-rim dinitro-substituted and lower-rim ethyl-ester-substituted calix[4]arene molecules, crystallized from polar aprotic or protic solvents (Gruber *et al.*, 2006 ▸). The intermolecular interactions affect the pinched-cone conformation of the host calixarene, which determines the accommodation of the guest molecules, optimizing electrostatic interactions (Fig. 10 ▸) in the calix crater or among the high-mobility lower-rim substituents. The structures are morphotropic, all with similar unit-cell parameters and identical space-group symmetries (monoclinic, *P*2_1_/*n*). The host framework of the inclusion complexes is mediated by weak C—H⋯O and C—H⋯π interactions.

**Figure 11:**
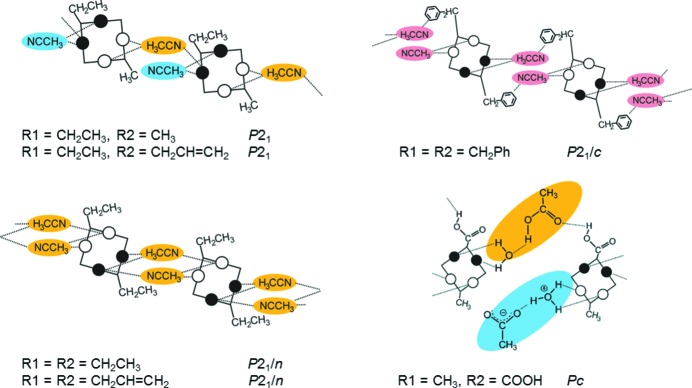
Synthon engineering: directed manipulation of the packing arrangement through supramolecular interactions. Examples from a series of laterally disubstituted calixarenes (Fischer *et al.*, 2012 ▸, 2013 ▸). The main packing motif in all structures is a molecular chain, irrespective of the size and chemical functionality of the lateral substituents, or the different space groups. The structural properties are sensitively tuned by application of secondary interactions governed by the fine balance of spatial and electrostatic forces.

The total space available for the guest molecules expands by the introduction of a lateral substituent to the calixarene molecule. The guest molecules are positioned interstitially as the cone conformation of the calixarene changes to a ‘partial cone’ by the effect of the bridge monosubstitution. The lateral attachment acts as a spacer, which at the same time reduces the close packing of the crystal due to increased asymmetry of the calixarene chalice. Influenced by the supramolecular interactions and the steric demands of the substituent, the serpentine-like channels observed in the unsubstituted calixarene structure are straightened to linear in the mono-substituted case (Fischer *et al.*, 2011 ▸). Introduction of a second substituent on the opposite methylene unit of the calix[4]arene exercises a distinct influence both on the host conformation and on the supramolecular architecture (Fig. 11 ▸). The partial cone transforms to a 1,2-alternate conformation and the two lateral attachments enforce a staggered arrangement of the calixarene molecules. This packing motif, a molecular chain along the axis of the bridge substitution, can be very sensitively tuned by the size and functionality of the substituents. The principal motif of the molecular column remains in the structures, irrespective of the size and functionality of the lateral substituents and the crystal system or space group. Nevertheless, the supramolecular synthon within the molecular chain might be tuned by the fine balance of the spatial and electrostatic forces (Fig. 11 ▸). The columnar packing motif appears even without guest molecules in the case of appropriately selected lateral substituents (Fischer *et al.*, 2012 ▸), and the chain is assembled irrespective of the polar or nonpolar character of the bridge substituent. If a size limit is exceeded, the lateral substituent takes part in the supramolecular bonding pattern. Keeping the robust common columnar packing motif, the crystal packing transforms from one pattern into another by translation and rotation in the different crystal structures. These calix[4]arene inclusion compounds are excellent examples of morphotropy induced by supramolecular interactions. The mastering of the supramolecular packing architecture, *e.g.* directed manipulation of molecular packing arrangement *via* the supramolecular interactions, can be appropriately described as *synthon engineering* (Bombicz *et al.*, 2014 ▸).

### 2.4. Kinetic assembly of coordination polymers   

Coordination polymers (also known as MOFs or coordination networks) have been actively investigated over the past quarter of a century because of the programmability of their architecture based on self-assembly of metal ions with bridging ligands (Hoskins & Robson, 1989 ▸, 1990 ▸; Fujita *et al.*, 1994 ▸; MacGillivray *et al.*, 1994 ▸; Yaghi & Li, 1995*a* ▸,*b* ▸; Kondo *et al.*, 1997 ▸; Millange *et al.*, 2002 ▸). The predictability of these self-assembled structures is attributed to the directionality of coordination bonds. During self-assembly, a global minimum can be achieved under thermodynamic conditions by regular repetition of reversible coordination bonding. Because of their stability and predictability, most researchers have studied thermodynamic products for various applications. However, unanticipated structures are often obtained as kinetic products (Gándara *et al.*, 2009 ▸). These have the potential to open up new fields in coordination polymer science, because kinetic products generally have more active pores. Moreover, through a kinetic product as an intermediate, a new thermodynamic product may be generated depending on experimental conditions. Many excellent reviews of coordination polymers have been written (Robson, 2008 ▸; Eddaoudi *et al.*, 2001 ▸; Furukawa *et al.*, 2013 ▸; Kitagawa *et al.*, 2004 ▸; Férey, 2008 ▸; Bétard & Fischer, 2012 ▸; Farha & Hupp, 2010 ▸; Cook *et al.*, 2013 ▸). This section will focus on recent progress on kinetic coordination polymers in which supramolecular interactions play a crucial role.

#### 2.4.1. Strategy for preparing kinetic coordination polymers   

**Figure 12:**
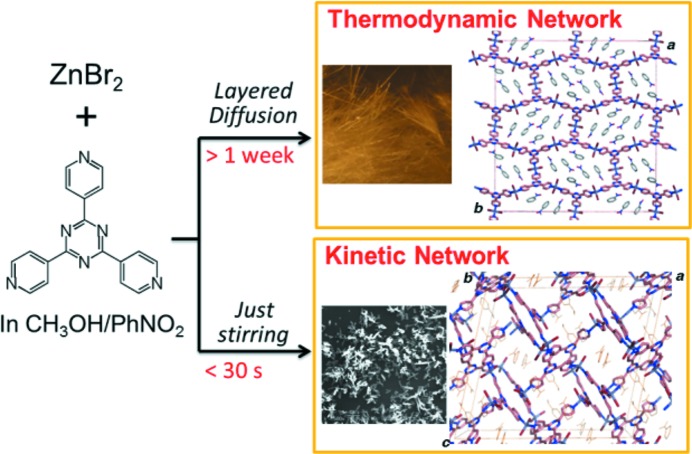
Selective network formation by thermodynamic/kinetic control.

The energy landscape for self-assembly of metal ions and bridging ligands can pass through many local minima before the global minimum is reached. The local minimum structure can be stabilized by various weak intermolecular interactions, such as π⋯π, CH⋯π, hydrogen bonds, halogen bonds *etc*. How can the formation of a kinetic coordination polymer be controlled? One of the simplest ways is to control temperature (Mahata *et al.*, 2008 ▸; Cheetham *et al.*, 2006 ▸). Depending on the activation energy, kinetic states can be trapped at appropriate temperatures. In addition, we can control the networking speed (reaction rate) to trap kinetic products. Our first finding of selective kinetic network formation was the reaction of ZnBr_2_ with TPT [2,4,6-tris(4-pyridyl)triazine] in nitrobenzene/methanol (Kawano *et al.*, 2008 ▸). Using the same starting materials and solvent, we prepared two types of porous network structures selectively in >50% yields (Fig. 12 ▸), depending on the reaction time (one week *versus* 30 s). These two structures have the same chemical formula, [(ZnBr_2_)_3_(TPT)_2_]·(solvent), and can therefore be considered to be polymorphs. Because of the extended nature of the structure, the local minima during self-assembly are much deeper than those of molecular crystals. Therefore, the kinetic network can be more readily trapped. However, a typical problem that arises while studying kinetic products is structure determination, because kinetic products often can be obtained only as fine crystalline powders. Although we could not determine a single-crystal structure of the [(ZnBr_2_)_3_(TPT)_2_]·(solvent) kinetic network, we succeeded in solving the structure using X-ray powder diffraction (XRPD) data. Structure determination from XRPD data can be a high hurdle for the study of kinetic networks, because the materials are relatively fragile and the unit-cell volume often can be over 10 000 Å^3^. The fragile nature of the materials can prohibit grinding to prepare uniform samples, thereby introducing difficulties associated with preferred orientation, while the large unit cell causes severe overlap of diffraction peaks. These technical difficulties could be one of the reasons why studies of kinetic networks have not yet been fully explored. For [(ZnBr_2_)_3_(TPT)_2_]·(solvent), we overcame these difficulties by developing the sample preparation method, *instant synthesis*, and by using synchrotron radiation.

Our strategy for preparing kinetic networks is based on structural information. Structural comparison of porous networks indicates the following features of a kinetic network: (1) it has fewer intermolecular interactions within a framework; (2) it tends to have larger void space; (3) it has more interactive sites in a pore, compared with a thermodynamically more stable network. Feature (3) is a natural consequence of (1), and it is important in that it can form a basic strategy to obtain an interactive pore without using elaborate methods, such as post-synthetic ones. Although a kinetic process can be intractable, we attempted to trap kinetic networks using multi-interactive ligands.

#### 2.4.2. Design of a multi-interactive ligand   

**Figure 13:**
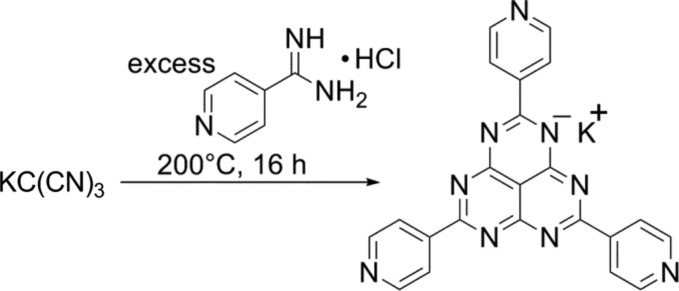
Preparation of multi-interactive ligand, K^+^·TPHAP^−^.

Introduction of interactive sites into ligands can deepen a local potential minimum by intermolecular interactions which will lead to trapping of kinetic states during self-assembly. Therefore, we designed a tridentate ligand with a radially extended character based on the hexaazaphenalene skeleton, TPHAP [2,5,8-tri(4-pyridyl)-1,3,4,6,7,9-hexaazaphenalene] (Yakiyama *et al.*, 2012 ▸). The potassium salt of TPHAP (K^+^·TPHAP^−^) can be synthesized on a gram scale by a simple one-pot reaction with a moderate yield (Fig. 13 ▸). The TPHAP^−^ anion has the following features: (*a*) a large aromatic plane for π–π interaction; (*b*) nine N atoms for hydrogen-bond or coordination-bond formation; (*c*) a delocalized negative charge over the central skeleton for charge-transfer interaction with guest molecules; (*d*) remarkable thermal stability of a single crystal of K^+^·TPHAP^−^, up to 500°C under an N_2_ atmosphere.

#### 2.4.3. Kinetic control of TPHAP network formations   

**Figure 14:**
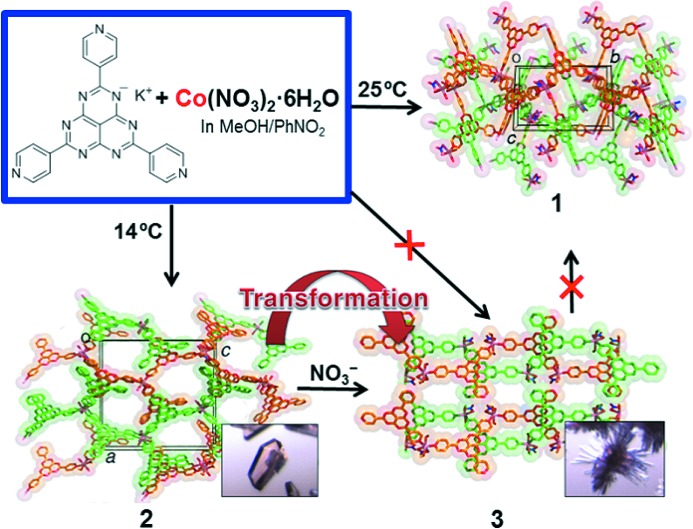
Selective Co-TPHAP^−^ network formation.

Reaction of K^+^·TPHAP^−^ and Co^II^ ions in a methanol/nitrobenzene solution generates several networks depending on temperature (Fig. 14 ▸). The reaction at 25°C generated thermally stable [Co(NO_3_^−^)(TPHAP^−^)(CH_3_OH)]·(solvent) (**1**), in which all TPHAP^−^ anions function as tridentate ligands to form a two-dimensional layered structure. However, using the same starting materials and solvent, the reaction at 14°C generated a totally different porous network, [Co(TPHAP^−^)_2_(CH_3_OH)_2_(H_2_O)]·(solvent) (**2**), which is a kinetic product. The network structure **2** is formed by hydrogen bonds and π–π stacking of one-dimensional chains composed of Co^II^ and both monodentate and bidentate TPHAP^−^. The Co^II^ centre has octahedral geometry coordinated by two bidentate and one monodentate TPHAP^−^, two methanol molecules, and one water molecule. Within a few days after the crystal formation, the crystals of **2** started to shrink and new dark-red crystals grew on the crystal surface. Synchrotron single-crystal X-ray analysis revealed that those crystals are uniform and the crystal structure is the coordination network, [Co(NO_3_^−^)(TPHAP^−^)(CH_3_OH)_2_]·(solvent) (**3**), com­posed of one-dimensional chains of Co^II^ and bidentate TPHAP^−^. During the crystal transformation, monodentate TPHAP^−^ and water around Co^II^ in **2** were exchanged with bidentate NO_3_^−^ to produce **3**. Crystal **3** showed no further transformation, indicating that **3** is thermodynamically more stable than **2**. Of note is that **3** can be prepared by structural transformation of **2**, but not through **1**. This illustrates that some materials can be prepared only *via* kinetic states rather than through thermodynamic experimental conditions.

#### 2.4.4. Diversity of TPHAP coordination networks   

**Figure 15:**
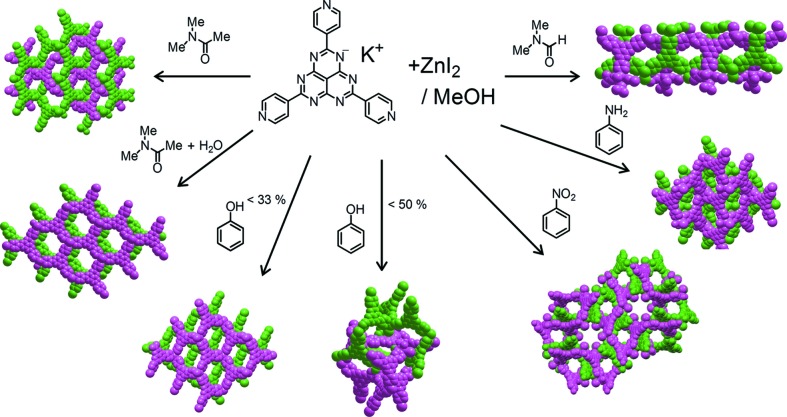
Diversity of TPHAP coordination networks.

As anticipated, the multi-interactive character of TPHAP^−^ allows many networks to be prepared from TPHAP^−^ and the same metal ions. For example, using ZnI_2_ and TPHAP^−^, seven kinds of networks were prepared depending on the nature of the solvent (Fig. 15 ▸) (Kojima *et al.*, 2014 ▸). Four of them consist of the same compound, ZnI(TPHAP^−^), and it is notable that two kinds of networks were obtained from the same solvent system (PhOH/MeOH), but with a different solvent ratio. This fact indicates that a very slight difference in experimental conditions can make a significant difference to the resulting network, presumably on account of the multi-interactivity of TPHAP^−^ and to weak intermolecular interactions. Furthermore, this extreme multi-interactivity can generate two kinds of pores, surrounded either by a π plane or by iodide, which can be expected to result in different selectivity for guest exchange.

#### 2.4.5. Formation of a stable network from a kinetic network   

**Figure 16:**
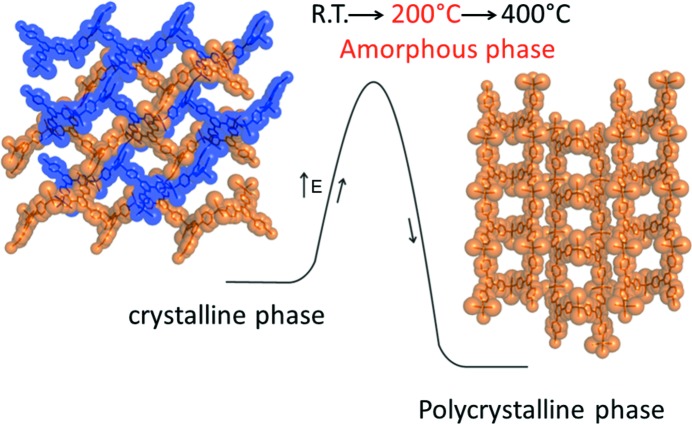
Crystalline-to-amorphous-to-crystalline network transformation.

As mentioned above, some materials can be prepared only through a kinetic intermediate by controlling the reaction conditions (Ohara *et al.*, 2009 ▸). The thermodynamic stability of a network is important for industrial applications. For example, a metastable interpenetrating network, [(ZnI_2_)_3_(TPT)_2_]·(solvent) (Biradha & Fujita, 2002 ▸), can be prepared as a fine powder by instant synthesis from ZnI_2_ and TPT in nitrobenzene/methanol. When the powder of the interpenetrating network is heated, an amorphous phase is generated at 200°C. Surprisingly, further heating at 300°C generates a new crystalline phase which is a saddle type of porous network (Fig. 16 ▸). The crystal structure was solved *ab initio* from XRPD data. The saddle structure is remarkably stable up to 400°C. It is noteworthy that it cannot be prepared by grinding and heating a mixed powder of ZnI_2_ and TPT. The fact that it is generated only *via* the interpenetrating structure indicates that preorganization of ZnI_2_ and TPT is essential.

**Figure 17:**
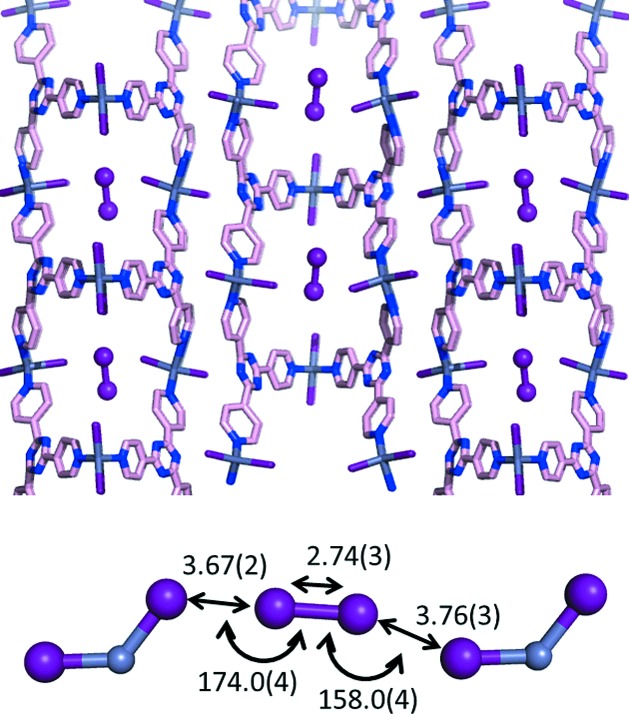
Crystal structure of I_2_ encapsulating the saddle network and the halogen–halogen interaction between I_2_ and ZnI_2_. Distances are given in Å and angles in degrees.

The saddle structure has a pore which can encapsulate small molecules, such as nitrobenzene, cyclohexane or I_2_. All of the guest-encapsulating network structures were solved by *ab initio* XRPD analysis (Martí-Rujas *et al.*, 2011 ▸). Encapsulation of I_2_ is particularly intriguing in that I⋯I interactions between ZnI_2_ and I_2_ play a crucial role in the encapsulation (I⋯I_2_ distance and angle: 3.67 (2) Å, 178.0 (4)° and 3.76 (3) Å, 158.0 (4)°; I—I bond length, 2.74 (3) Å, Fig. 17 ▸). The geometry indicates a typical halogen–halogen interaction between positive (σ-hole) and negative sites (unshared electron). The geometry that we observed is comparable to an earlier reported structure of I_2_ in an encapsulating network (Lang *et al.*, 2004 ▸).

#### 2.4.6. Molecular crystalline flasks: direct observation of a reactive sulfur allotrope in a pore   

**Figure 18:**
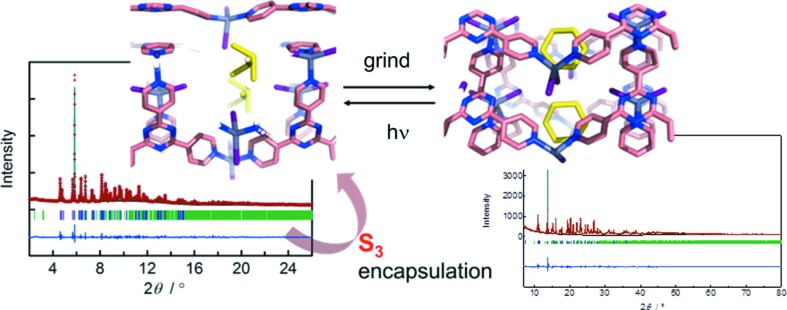
Selective S_3_ trapping in a pore of saddle structure and the reversible conversion into S_6_.

Crystalline porous networks can function as nanoscale molecular flasks (Kawamichi *et al.*, 2008 ▸; Inokuma *et al.*, 2011 ▸) that allow *in situ* crystallographic monitoring of guest encapsulation (Matsuda *et al.*, 2005 ▸; Kawano & Fujita, 2007 ▸), reactive species (Ohtsu *et al.*, 2013 ▸) or chemical reactions (Kawamichi *et al.*, 2009 ▸) within a pore. We attempted to trap reactive small allotropes of sulfur into a pore of the saddle-structure crystal by sulfur vapour diffusion at 260°C for 6 h under vacuum. After exposure to sulfur vapour, the powder turned from pale yellow to bright yellow and the XRPD pattern clearly changed. *Ab initio* XRPD analysis revealed that S_3_ is selectively trapped in a pore (Fig. 18 ▸). This is the first crystal structure determination of a reactive sulfur allotrope smaller than S_6_. The open-triangle *C*_2*v*_ structure of S_3_ is in good agreement with structures obtained by rotational spectroscopy.

Although S_3_ is an ozone analogue, TG–DSC revealed that it can remain in the pore until around 230°C. From the above-mentioned I_2_ encapsulation study, the iodide of ZnI_2_ in the saddle structure can be considered as an interactive site in the pore, and S_3_ is stabilized by intermolecular interactions with iodide in the pore. However, we found that S_3_ can be converted into S_6_ by grinding at room temperature or by heating in the presence of NH_4_ *X* (*X* = Cl, Br). The S_6_ ring is also involved in intermolecular interactions with iodide in the pore. Furthermore, S_6_ can be reversibly converted into S_3_ by UV irradiation.

Kinetic assembly can generate larger and more active pores compared with thermodynamic assembly. Using a rigid *T*_d_ symmetry ligand TPPM [tetra(4-(4-pyridyl)phenyl)methane] and a kinetically labile CuI unit, we have recently succeeded in selective preparation of thermodynamic and kinetic porous networks having an active pore, which show unique physi- and chemisorption of I_2_, respectively (Kitagawa *et al.*, 2013 ▸). Our findings promise that interactive crystalline pores can provide an unprecedented opportunity to adsorb unique guest molecules and to observe chemical reactions stepwise by crystallographic techniques.

## 3. Conclusion   

This article has discussed four topics within the vast scope of supramolecular interactions in the solid state. They are tied together by the broad research field of crystal engineering, and we feel that the discussion touches upon many of the prerequisites for an improved understanding of preparing and characterizing crystalline materials. The historical overview of halogen bonding illustrates how development of our understanding of the fundamental nature of supramolecular interactions enables unification of seemingly diverse chemical observations, and permits these interactions to be exploited in strategies for solid-state materials design. The balance between intermolecular interactions in the solid state is reflected as snap-shots within isostructural, morphotropic and polymorphic crystal structures, and can be investigated in more depth by structural characterization under non-ambient conditions. Since the relative inputs of different types of interactions to the structure stability can vary with temperature and pressure, this understanding should present new prospects for polymorphism control and crystal engineering, making it possible to obtain and preserve new crystalline forms either in stable or metastable states. The strategic design of metastable coordination networks illustrates already that this is possible, and the resulting interactive crystalline pores provide exciting opportunities to observe new chemical reactions by crystallographic techniques.

## Figures and Tables

**Figure 2:**
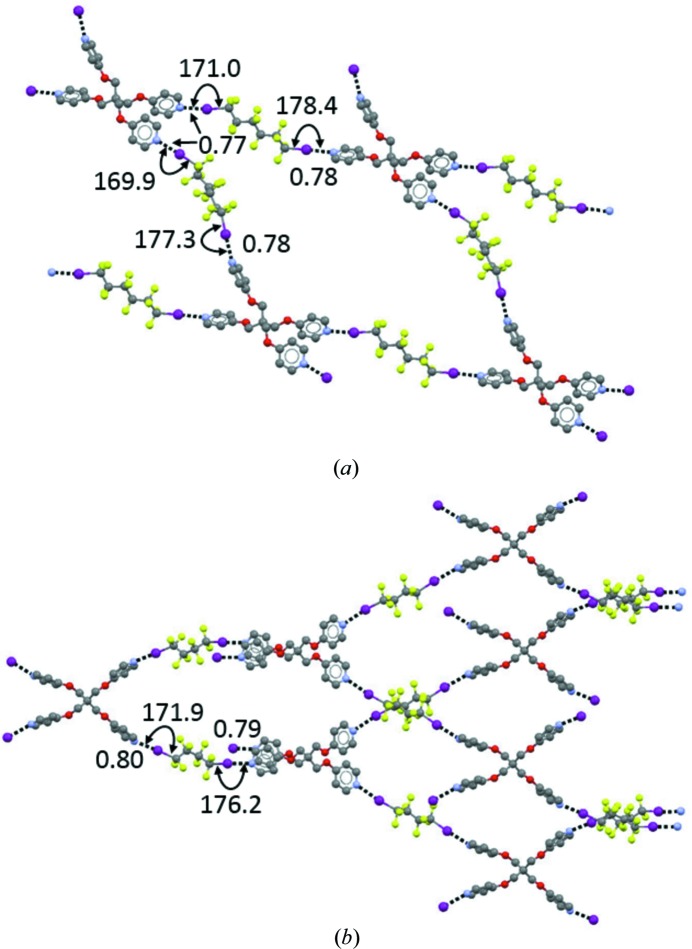
Halogen-bonded adducts formed by the tetradentate XB acceptor tetra-4-pyridyl-pentaerythritol. (*a*) Two-dimensional square grid formed with the bidentate XB donor 1,6-diiodoperfluorohexane; (*b*) three-dimensional adamantanoid network formed with the bidentate XB donor 1,4-diiodoperfluorobutane (Metrangolo *et al.*, 2007 ▸). H atoms are omitted. XBs are shown as black dotted lines. XB separations, angles and colour codes are as in Fig. 1 ▸.
